# Aberrant Activation of p38 MAP Kinase-Dependent Innate Immune Responses Is Toxic to *Caenorhabditis elegans*

**DOI:** 10.1534/g3.115.025650

**Published:** 2016-01-27

**Authors:** Hilary K. Cheesman, Rhonda L. Feinbaum, Jose Thekkiniath, Robert H. Dowen, Annie L. Conery, Read Pukkila-Worley

**Affiliations:** * Program in Innate Immunity, Division of Infectious Diseases and Immunology, University of Massachusetts Medical School, Worcester, Massachusetts 01605; † Department of Molecular Biology, Massachusetts General Hospital, Boston, Massachusetts 02114

**Keywords:** innate immunity, immune regulation, *C. elegans* genetics, host-pathogen interactions, genetics of immunity

## Abstract

Inappropriate activation of innate immune responses in intestinal epithelial cells underlies the pathophysiology of inflammatory disorders of the intestine. Here we examine the physiological effects of immune hyperactivation in the intestine of the nematode *Caenorhabditis elegans*. We previously identified an immunostimulatory xenobiotic that protects *C. elegans* from bacterial infection by inducing immune effector expression via the conserved p38 MAP kinase pathway, but was toxic to nematodes developing in the absence of pathogen. To investigate a possible connection between the toxicity and immunostimulatory properties of this xenobiotic, we conducted a forward genetic screen for *C. elegans* mutants that are resistant to the deleterious effects of the compound, and identified five toxicity suppressors. These strains contained hypomorphic mutations in each of the known components of the p38 MAP kinase cassette (*tir-1*, *nsy-1*, *sek-1*, and *pmk-1*), demonstrating that hyperstimulation of the p38 MAPK pathway is toxic to animals. To explore mechanisms of immune pathway regulation in *C. elegans*, we conducted another genetic screen for dominant activators of the p38 MAPK pathway, and identified a single allele that had a gain-of-function (gf) mutation in *nsy-1*, the MAP kinase kinase kinase that acts upstream of p38 MAPK *pmk-1*. The *nsy-1(gf)* allele caused hyperinduction of p38 MAPK PMK-1-dependent immune effectors, had greater levels of phosphorylated p38 MAPK, and was more resistant to killing by the bacterial pathogen *Pseudomonas aeruginosa* compared to wild-type controls. In addition, the *nsy-1(gf)* mutation was toxic to developing animals. Together, these data suggest that the activity of the MAPKKK NSY-1 is tightly regulated as part of a physiological mechanism to control p38 MAPK-mediated innate immune hyperactivation, and ensure cellular homeostasis in *C. elegans*.

Coordination of innate immune responses at mucosal surfaces is a critical determinant of cellular homeostasis in evolutionarily diverse organisms (Peterson and Artis 2014). Exaggerated or aberrantly triggered immune responses, for example, underlie the pathophysiology of inflammatory disorders of the human intestine (Xavier and Podolsky 2007). In flies, immune pathway hyperactivation also has negative physiological consequences and is subject to feedback control (Aggarwal and Silverman 2008).

To understand ancient mechanisms of pathogen detection and immune regulation, our group and others are examining innate immune responses in the nematode *Caenorhabditis elegans* (Irazoqui *et al.* 2010; Pukkila-Worley and Ausubel 2012; Cohen and Troemel 2015). As in other metazoans, nematodes coordinate inducible immune defenses from intestinal epithelial cells (IECs), which provide a critical barrier against ingested pathogens (Kim *et al.* 2002; Shivers *et al.* 2009; Pukkila-Worley *et al.* 2011). Studies from *C. elegans* and other diverse organisms have revealed that key innate immune signaling regulators are strongly conserved (Irazoqui *et al.* 2010; Pukkila-Worley and Ausubel 2012; Visvikis *et al.* 2014; Cohen and Troemel 2015). For example, the NSY-1-SEK-1-PMK-1 Mitogen Activated Protein Kinase (MAPK) pathway in *C. elegans*, which is orthologous to the mammalian ASK1-MKK3/6-p38 MAPK pathway, controls the induction of putative antimicrobial immune effectors, and is required in intestinal epithelial cells for nematodes to survive challenge from ingested pathogens (Kim *et al.* 2002; Troemel *et al.* 2006; Shivers *et al.* 2010; Pukkila-Worley *et al.* 2011). In mammals, the ASK1-MKK3/6-p38 signaling cassette is a central regulator of inflammatory cytokine production in response to pathogen detection at epithelial surfaces, and pathway activation is tightly regulated through negative regulatory circuits (Kyriakis and Avruch 2012). Misregulation of p38 signaling in IECs has been implicated in the pathogenesis of inflammatory bowel disease, cancer, autoimmune disorders, and immunodeficiency syndromes (Waetzig *et al.* 2002; Kyriakis and Avruch 2012). From an evolutionary perspective, it is therefore logical that mechanisms of immune homeostasis are selected for as part of a survival strategy, particularly for organisms such as bacterivorous nematodes that live in microbe-rich environments and must distinguish pathogens from potential food sources.

Here we focus on the p38 MAPK pathway in *C. elegans*, and examine the physiological consequences of innate immune hyperactivation in intestinal epithelial cells. From two different forward genetic screens, we present evidence that aberrant activation of p38 MAPK-mediated defenses in intestinal epithelial cells is deleterious to nematodes. We found that the toxicity associated with exogenous stimulation of the p38 MAPK-mediated immune responses can be suppressed through loss-of-function mutations in p38 MAPK pathway components. We also identify and characterize a gain-of-function allele of the MAPKKK *nsy-1* that drives hyperactivation of the p38 MAPK PMK-1 pathway. Accordingly, the *nsy-1(gf)* allele is protective against bacterial infection, but the concomitant hyperactivation of innate immune effectors is deleterious to developing animals. These data suggest that the MAPKKK NSY-1 is negatively regulated as a part of mechanism to ensure immune homeostasis.

## Materials and Methods

### C. elegans and bacterial strains

**Figure 1:**
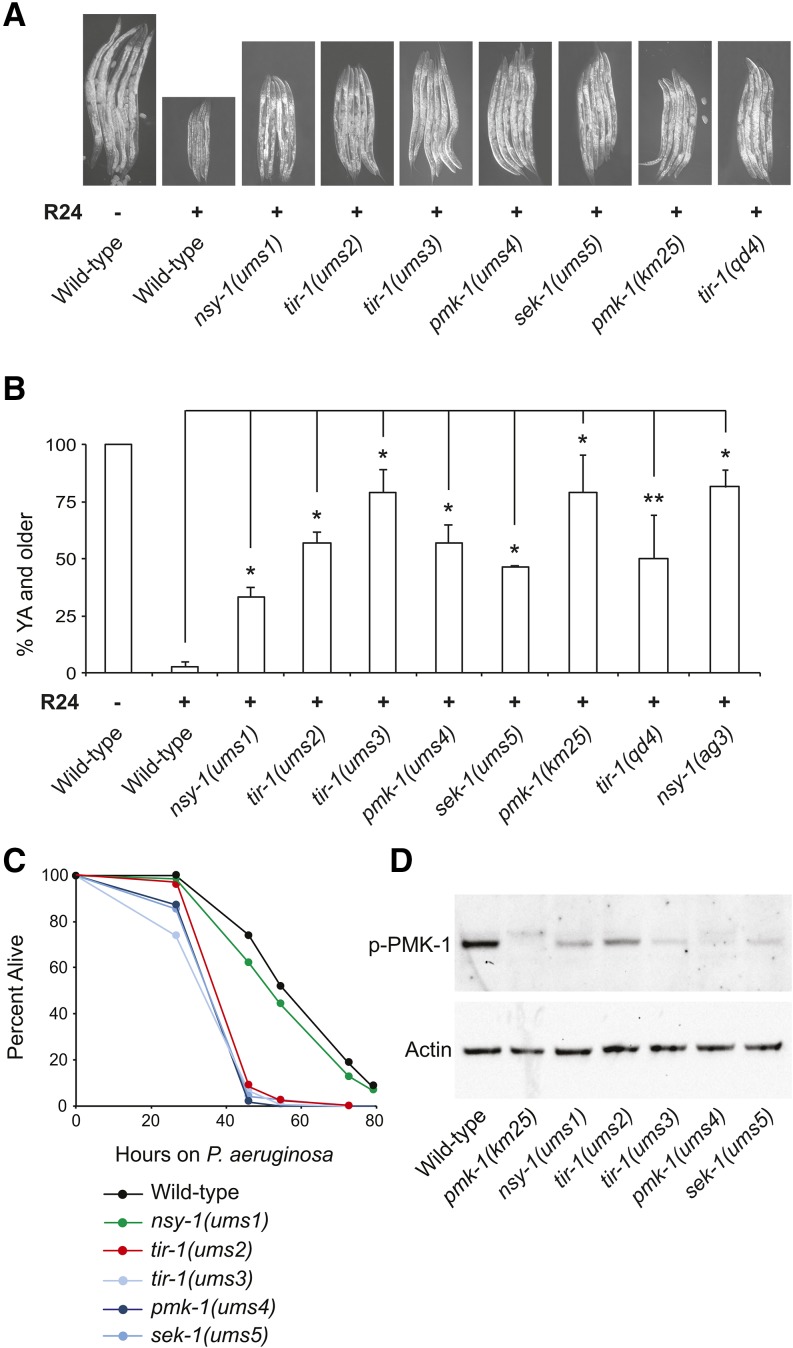
p38 MAPK mutants have a Xenobiotic toxicity suppressor (Xts) phenotype. (A) Representative images of *C. elegans* mutants with an Xts phenotype photographed after 3 d of development at 20°C in the presence (+) or absence (–) of 140 μM R24. *C. elegans* N2 animals were used as the wild-type control strain. (B) Quantification of the percentage of animals that grew from the L1 to young adult (YA) stage for the experiment shown in (A). Data are the average of two technical replicates, with error bars giving the standard deviation between plates. The sample sizes for this experiment are: N2 (196), *nsy-1(ums1)* (171), *tir-1(ums2)* (193), *tir-1(ums3)* (220), *pmk-1(ums4)* (145), *sek-1(ums5)* (196), *pmk-1(km25)* (155), *tir-1(qd4)* (269) and *nsy-1(ag3)* (193). * *P* < 0.05, ** *P* = 0.07. The data are representative of multiple biological replicate experiments, which were conducted during the backcrossing of the *ums* mutants to wild-type animals. (C) A *P. aeruginosa* pathogenesis assay with *C. elegans* wild-type N2 and mutants with an Xts phenotype is shown. The difference in *P. aeruginosa* susceptibility between all mutants with an Xts phenotype and wild-type animals is significant (*P* < 0.001), except *nsy-1(ums1)* (*P* = n.s.). Sample sizes are: wild-type N2 (152), *nsy-1(ums1)* (140), *tir-1(ums2)* (151), *tir-1(ums3)* (150), *pmk-1(ums4)* (124), and *sek-1(ums5)* (139). (D) Immunoblot analysis of lysates from L4 larval stage animals of the indicated genotype using antibodies that recognize the doubly phosphorylated TGY motif of PMK-1 (p-PMK-1) and actin.

*C. elegans* was grown and propagated on NGM plates with *Escherichia coli* OP50, as described (Brenner 1974). AU307, an N2 Bristol-derived strain carrying the *agIs44* transgene (*pF08G5.6*::*GFP*::*unc-54-3′UTR*; *pmyo-2*::*mCherry*), which was outcrossed to the wild-type strain N2 five times (Pukkila-Worley *et al.* 2014), was used as the wild-type strain for these studies, unless otherwise noted. N2 was used as the wild-type strain for the studies presented in Figure 1. Previously isolated and characterized mutants used in this study were: *nsy-1(ag3) II* (Kim *et al.* 2002), *tir-1(qd4) III* (Shivers *et al.* 2009), *xbp-1(zc12) III; zcIs4 V* (Calfon *et al.* 2002), *pmk-1(km25) IV* (Kim *et al.* 2002), and *zcIs4 V* (Calfon *et al.* 2002). RPW1 *nsy-1(ums1) II*, RPW2 *tir-1(ums2) III*, RPW3 *tir-1(ums3) III*, RPW4 *pmk-1(ums4) IV*, and RPW5 *sek-1(ums5) X* were isolated in this study as described below. RPW43 *agIs44*; *nsy-1(ums8)* was also isolated in this study as described below, and outcrossed six times to the wild-type strain N2.

### Isolation and identification of mutants with an Xts phenotype

Mutagenesis of wild-type N2 animals was performed with ethyl methane sulfonate (EMS, Sigma-Aldrich Co.) following standard methods (Jorgensen and Mango 2002). In two separate rounds of EMS screening, F2 animals from approximately 100,000 mutagenized haploid genomes were synchronized by hypochlorite treatment, and plated onto “slow kill” media (Tan *et al.* 1999) containing 140 μM of R24. We previously used 70 μM for our characterization of R24 immunostimulatory properties (Pukkila-Worley *et al.* 2012). The lower concentration also delayed the development of wild-type nematodes, but the phenotype was more pronounced at a concentration of 140 μM. Animals that reached at least the L4 larval stage after approximately 60 hr at 20° were singled to a new plate. Of the unmutagenized animals treated in parallel, 100% were at the L2 or L3 larval stage at this time point. Five mutants with the most penetrant Xts phenotypes after three rounds of retesting were identified using next-generation sequencing technology (Illumina, Inc.) following established methods (Sarin *et al.* 2008). Briefly, the progeny from individual F2 recombinant animals that had the mutant phenotype following a 1X backcross to N2 animals were pooled for *ums3*, *ums4*, and *ums5* (40, 38, and 33 pooled recombinants, respectively). DNA was isolated from these samples, and from the original *ums1* and *ums2* mutants, using the Gentra Puregene Kit (Qiagen). Libraries for deep sequencing were prepared using the NEBNext DNA Library Prep Reagent Set and Oligos (New England Biolabs, Inc.), and sequenced using Illumina HiSeq 2500 following instructions from the manufacturer. Homozygous variants from the WS220 (ce10) *C. elegans* reference genome were identified using CloudMap (Minevich *et al.* 2012), and removed from further analysis if they were present in the unmutagenized parent wild-type N2 strain, the genome of which was also sequenced.

### Isolation and identification of a constitutive activator of F08G5.6::GFP

EMS mutagenesis was performed on strain *agIs44* as described above. Synchronized F1 progeny from approximately 170,000 mutagenized haploid genomes were screened for animals that constitutively express *agIs44* GFP (green fluorescent protein) fluorescence using a dissecting microscope that is able to visualize GFP. A single mutant allele, *ums8*, was identified. To identify the *ums8* mutation, DNA was isolated and sequenced using the methods described above. Pools of progeny from a total of 52 individual recombinants from a single outcross to wild-type N2, and from 43 individual recombinants that were outcrossed twice to N2, were sequenced. All recombinants selected for sequencing expressed *agIs44* GFP fluorescence constitutively. Homozygous variants from the WS220 (ce10) *C. elegans* reference genome that were present in both the 1X and 2X backcross samples, but were not present in the parent *agIs44* strain, were identified using CloudMap (Minevich *et al.* 2012).

### RNAi of nsy-1

Two RNAi constructs were created using 731 bp and 1007 bp segments of *nsy-1* coding region (bases 32,533 to 33,264, and 30,950 to 31,957 on cosmid *F59A6*, respectively), which were amplified by PCR and subcloned into the Fire vector pPD129.36, ligation number L4440 (referred to throughout the manuscript as L4440) to create plasmids pHC1 and pHC2, respectively. These plasmids were transformed into the RNAi bacterial feeder strain HT115, and RNAi experiments were carried out with these strains following established protocols (Timmons *et al.* 2001) using HT115 bacteria expressing the empty vector L4440 as the control. For all RNAi experiments, L4 larval stage animals of the indicated genotypes were picked to RNAi bacteria, and F1 progeny were used for subsequent studies, as described in more detail below.

### nanoString nCounter gene expression and quantitative RT-PCR (qRT-PCR)

For the nanoString transcription profiling experiment, hypochlorite-synchronized L1 larval stage animals were added to NGM plates seeded with OP50. *nsy-1(ums8)* mutant animals were added to these plates approximately 24 hr before the *nsy-1(ag3)* and *agIs44* animals, to allow the animals to reach the L4 larval stage at the time of harvest. The *agIs44* animals were used as the wild-type control for this experiment. Worms were flash-frozen in an ethanol and dry ice bath, lysed in 0.5% SDS, 5% β-mercaptoethanol, 10 mM EDTA, 10 mM Tris-HCl pH 7.4., 0.5 mg/ml Proteinase K at 55° for 15 min using a previously described protocol (Ding *et al.* 2015), RNA was isolated using Tri-reagent (Sigma-Aldrich Co.), and 100 ng was analyzed by the nanoString nCounter Gene Expression System (nanoString Technologies, Inc.) using a “codeset” designed by nanoString that contained probes for 118 *C. elegans* genes (Supporting Information, Table S1). Probe hybridization, data acquisition and analysis were performed according to instructions from nanoString with the expression data from each sample normalized to the geometric mean of expression values for the control genes *snb-1*, *ama-1*, and *act-1*.

To determine the gene induction in *nsy-1(ums8)/+* heterozygotes, RNA was isolated from 100 L4 larval stage *agIs44* (+/+), *nsy-1(ums8)/nsy-1(ums8)* homozygotes, and *nsy-1(ums8)/+* heterozygotes, the latter of which were the F1 progeny from a cross between *agIs44* animals and *nsy-1(ums8)* homozygotes. RNA was isolated from three replicates using Trizol (Sigma-Aldrich Co.), treated with recombinant DNaseI (Ambion), reverse transcribed to cDNA using the Retro-script kit (Life Technologies), and analyzed using iQ SYBR Green detection (Bio-Rad Laboratories, Inc.) in duplicate 20-μl reactions on a CFX1000 machine (Bio-Rad Laboratories, Inc.) with previously published primers (Troemel *et al.* 2006; Pukkila-Worley *et al.* 2012). All values were normalized against the control gene *snb-1*. Fold change was calculated using the Pfaffl method (Pfaffl 2001). For the other qRT-PCR studies, four L4 larval stage animals of the indicated genotype were added to RNAi bacteria, and RNA was harvested from the mixed-stage F1 progeny from these animals. To ensure that animals would be approximately stage-matched at the time of harvest, *nsy-1(ums8)* mutants were allowed to lay their brood and develop at 20°, while *agIs44* and *nsy-1(ag3)* animals were kept at 15°. RNA was isolated from three replicates using Tri-reagent (Sigma-Aldrich Co.), reversed transcribed into cDNA, and studied by qRT-PCR following the protocol described above.

### Immunoblot analyses

*C. elegans* was prepared in the manner described for the nanoString experiment to ensure that stage-matched animals at the young L4 larval stage were studied in each condition. A previously described protocol (Ding *et al.* 2015) was adapted for the immunoblot analysis. Harvested animals were washed twice with M9 buffer, incubated in a roller at room temperature for 15 min to allow the nematode intestine to clear of bacteria, washed an additional time and flash-frozen in RIPA Buffer (Cell Signaling Technology, Inc.) using an ethanol and dry ice bath. Samples were lysed by sonication and centrifuged. Protein was quantified from the supernatant of each sample using Bradford Reagent (Bio-Rad Laboratories, Inc.). Laemmli buffer (Bio-Rad Laboratories, Inc.) was added to a concentration of 1X, and the total protein from each sample was resolved on NuPage 4–12% gels (Life Technologies), transferred to nitrocellulose membranes (Life Technologies), blocked with 5% powdered milk in TBST, and probed with a 1:2000 dilution of an antibody that recognizes the doubly phosphorylated TGY motif of PMK-1 (Promega Corporation). The blot was then stripped and reprobed with a 1:10,000 dilution of an anti-actin antibody (Thermo Fisher Scientific, Inc.). Horseradish peroxidase (HRP)-conjugated anti-rabbit, and anti-mouse IgG secondary antibodies (Thermo Fisher Scientific, Inc.) were used to detect the primary antibodies following the addition of ECL reagents (Thermo Fisher Scientific, Inc.), which were visualized using a Fujifilm LAS-400 luminescent image analyzer.

### C. elegans bacterial infection and development assays

“Slow killing” *P. aeruginosa* infection assays were performed as previously described (Tan *et al.* 1999). A single colony of *P. aeruginosa* PA14 was inoculated into 3 ml of Luria-Bertani (LB) medium, and allowed to incubate at 37° for 14–15 hr; 10 μl of this culture was added to 35-mm tissue culture plates containing 4 ml of slow kill agar (0.35% peptone, 0.3% sodium chloride, 1.7% agar, 5 μg/ml cholesterol, 25 mM potassium phosphate, 1 mM magnesium sulfate, 1 mM calcium chloride). Plates were incubated for 24 hr at 37°, and 24 hr at 25°. At 1–2 hr before the start of the assay, 0.1 mg/ml 5-fluorodeoxyuridine (FUDR) was added to the medium to prevent progeny from hatching. A total of 40–50 animals at the young L4 larval stage were picked to each of three or four assay plates per experimental condition. *C. elegans* was prepared for the pathogenesis assays in the manner described above for the nanoString experiment to ensure that stage-matched nematodes were used for these experiments. Animals were scored as live or dead on a daily basis by gently touching them with a platinum wire. Worms that crawled onto the wall of the tissue culture plate were eliminated from the analysis. *P. aeruginosa* killing assays were conducted at 25°.

The development assay presented in Figure 1 was conducted by placing approximately 100 hypochlorite-synchronized L1 larval stage animals of the indicated genotype on “slow kill” medium plates (Tan *et al.* 1999) containing 140 μM R24 or the solvent control DMSO (1%), and monitoring development to the young adult stage for 3 d at 20° on two replicate plates per condition. For the development assays conducted with RNAi bacteria (Figure 4B), two L4 larval stage animals of the indicated genotype were allowed to lay their brood on RNAi bacteria at 15°. Plates were then transferred to 20° for 3 d. The stage of approximately 300 animals on each of three replicate plates per condition was recorded, and the percentage of animals at the L4 larval stage was reported. Representative animals from each condition in this experiment were photographed. For the experiments with the *xbp-1(zc12)* mutant (Figure S4), four animals were allowed to lay their brood for 8 hr at 20° in the presence or absence of 70 μM R24. *C. elegans* carrying the *zcIs4* transgene was used as the control for these experiments.

### Microscopy

Nematodes were mounted onto 2% agar pads, paralyzed with levamisole (Sigma-Aldrich Co.), and photographed using a AXIO Imager Z1 microscope with a AxioCam HRm camera and Axiovision 4.6 software (Zeiss), or an Eclipse E400 with a DS-QilMc camera and NIS Elements Imaging Software (Nikon Corporation). Photographs were acquired using the same imaging conditions for a given experiment, and were processed in Photoshop (Adobe Systems, Inc.).

### Statistical analyses and amino acid alignment

Differences in survival of *C. elegans* animals in the *P. aeruginosa* pathogenesis assays were determined with the log-rank test. Fold changes in the qRT-PCR analyses and differences in the development of animals to the indicated stage were compared using unpaired, two-tailed student *t*-tests. Amino acid alignment between human ASK1, the *Drosophila* Pk92B, and *C. elegans* NSY-1, was determined using ClustalW2 (Larkin *et al.* 2007).

### Data availability

Strains are available upon request. Accession numbers for genes and gene products are given for the publically available database Wormbase (http://www.wormbase.org). The accession numbers for the principal genes mentioned in this paper are: *C17H12.8*, *C32H11.12*, *F08G5.6*, *F35E12.5*, *F56D6.2*, *nsy-1 (F59A6.1)*, *pmk-1 (B0218.3)*, *sek-1 (R03G5.2)*, *tir-1(F13B10.1)*, *T24B8.5*. Other accession numbers are given in Table S1.

## Results and Discussion

### Exogenous hyperactivation of the p38 MAP kinase pathway is toxic to developing C. elegans

The small molecule R24, also called RPW-24, acts upstream of the p38 MAPK pathway to cause the induction of putative antibacterial immune effectors in the intestine of *C. elegans* and, accordingly, protects nematodes from bacterial infection (Pukkila-Worley *et al.* 2012, 2014). In addition, exposure to R24 causes the induction of detoxification enzymes and delays the development of wild-type nematodes in the absence of pathogen, suggesting that this compound is toxic to *C. elegans* under normal laboratory growth conditions (Pukkila-Worley *et al.* 2012, 2014). To investigate the relationship between the immunostimulatory properties and the toxicity of this anti-infective xenobiotic, we screened nematodes derived from approximately 100,000 mutagenized haploid genomes for mutants that were able to develop faster in the presence of 140 μM R24 (Figure 1, A and B). We chose this concentration because the developmental delay phenotype was more pronounced than observed for 70 μM R24, the concentration we had used in our previous studies (Pukkila-Worley *et al.* 2012, 2014). For the screen, we required the selected mutants to be L4 larval stage or older at a time point when 100% of unmutagenized worms treated with R24 in parallel were at the L2 or L3 larval stage. Seven mutants with a xenobiotic toxicity suppressor (Xts) phenotype were identified, and we focused on the five mutants that had the most penetrant Xts phenotypes which we have called *ums1*, *ums2*, *ums3*, *ums4* and *ums5* (Figure 1, A and B). To identify the mutation in these strains that permitted improved development in the presence of R24, we used next-generation sequencing technology (Sarin *et al.* 2008; Minevich *et al.* 2012). For three mutants (*ums3*, *ums4*, and *ums5*), we sequenced DNA from pooled F2 recombinants that had the mutant phenotype following a backcross to wild-type N2 animals, and for two mutants (*ums1* and *ums2*) we identified mutations after sequencing the original mutant strain. Interestingly, these five mutants each contained missense mutations in one of the four known components of the p38 MAPK signaling cassette (*tir-1*, *nsy-1*, *sek-1*, or *pmk-1*, Table 1).

**Table 1:** Xenobiotic toxicity suppressors

Gene	Allele	Mutation	Description, Mammalian Homolog
*tir-1*	*ums2*	L730F	TIR domain adaptor protein, SARM
*tir-1*	*ums3*	A723T	TIR domain adaptor protein, SARM
*nsy-1*	*ums1*	P874L	MAPKKK, ASK1
*sek-1*	*ums5*	G199S	MAPKK, MKK3/6
*pmk-1*	*ums4*	L296F	MAPK, p38

Presented is a list of isolates identified in a screen for mutants that are able to develop faster in the presence of the immunostimulatory anti-infective xenobiotic R24 compared to controls.

To characterize these newly-isolated p38 MAPK pathway mutants, we conducted pathogenesis assays with *P. aeruginosa* and found that, as with the classic loss-of-function mutants in p38 MAPK pathway components, four of the five mutants [*tir-1(ums2)*, *tir-1(ums3)*, *pmk-1(ums4)* and *sek-1(ums5)*] had an enhanced susceptibility to pathogens (Esp) phenotype (*P* < 0.001, Figure 1C). *nsy-1(ums1)*, the weakest toxicity suppressor of these five mutants, did not have an obvious Esp phenotype. In addition to conferring an Esp phenotype, loss- and reduction-of-function mutations in upstream components of the p38 MAPK PMK-1 pathway cause a reduction in the amount of activated PMK-1, which can be detected in an immunoblot experiment using an antibody that specifically recognizes the doubly phosphorylated TGY motif of PMK-1 (Kim *et al.* 2002; Liberati *et al.* 2004). We found that *nsy-1(ums1)*, *tir-1(ums2)*, *tir-1(ums3)*, *pmk-1(ums4)* and *sek-1(ums5)* each had reduced levels of the active form of PMK-1 compared to wild-type controls (Figure 1D). These data indicate that the forward genetic screen for mutations that confer resistance to the toxic effects of the immunostimulatory xenobiotic R24 identified hypomorphic alleles in the p38 MAPK pathway. Interestingly, a previous forward genetic screen for loss-of-function mutations in the p38 MAPK pathway components identified two alleles, *pmk-1*(*qd9*) and *sek-1(qd37)*, which had the identical missense mutation as *pmk-1(ums4)* and at the same amino acid as *sek-1(ums5)*, respectively (Shivers *et al.* 2010).

Together, these data demonstrate that the toxicity of R24 can be suppressed by hypomorphic mutations in the p38 MAPK pathway, although no single mutation was identified that resulted in growth progression identical to worms in the absence of the compound. To confirm this observation, we studied the previously characterized null alleles *tir-1(qd4)*, *nsy-1(ag3)* and *pmk-1(km25)* (Kim *et al.* 2002; Shivers *et al.* 2009) (Figure 1, A and B) and found that these mutations also suppressed the R24-induced developmental delay to a degree comparable to the *pmk-1(ums4)* mutant, and the *tir-1(ums2)* and *tir-1(ums3)* alleles, respectively (Figure 1, A and B).

In summary, these data indicate that hyperactivation of p38 MAPK immune defenses is toxic to developing nematodes.

### Forward genetic screen uncovers a gain-of-function allele in the MAPKKK nsy-1

**Figure 2:**
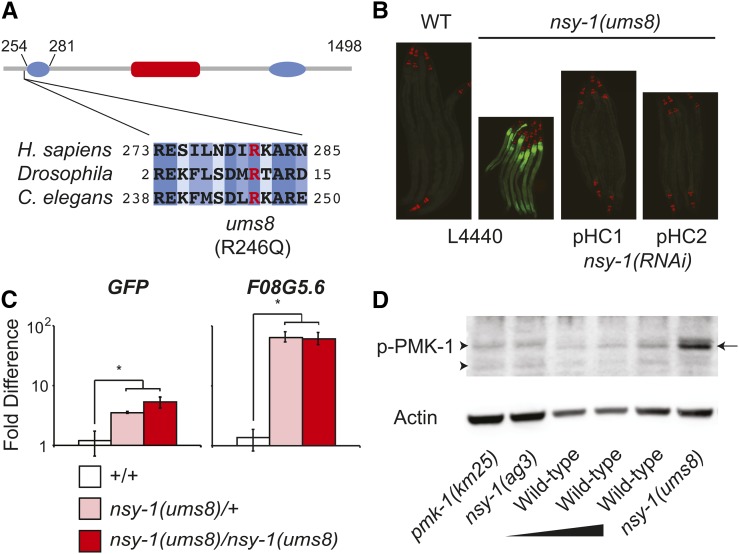
The *nsy-1(ums8)* allele encodes a gain-of-function mutation in *nsy-1*. (A) The putative domain architecture of NSY-1, based on homology to mammalian ASK1, is presented. This schematic was adapted from Bunkoczi *et al.* (2007). The putative location of the central serine-threonine kinase domain, and two coiled-coil domains in the N and C termini are shown in red and blue, respectively. The boundary of the N-terminal negative regulatory domain relative to the NSY-1 protein is presented above the diagram. The Arg that was mutated to Gln in the *ums8* strain (R246Q) is highlighted in red. Amino acid sequence alignment of human ASK1, *Drosophila* Pk92B (the ASK1 homolog) and *C. elegans* NSY-1 demonstrates that the *ums8* mutation is located in a strongly conserved region. Dark blue shading indicates identical amino acids, with progressive lighter blue shading indicating the level of similarity in amino acid class as determined by the software ClustalW2. (B) Wild-type (WT) and *nsy-1(ums8)* animals were exposed to the feeding RNAi bacteria strain transformed with the control vector (L4440), or with two separate RNAi constructs (pHC1 and pHC2) that target different areas of coding region in the *nsy-1* gene and photographed. Green is *F08G5.6*::*GFP* induction and red is *myo-2*::*mCherry*, which was used as the co-injection marker. (C) The expression of the indicated genes was determined using qRT-PCR in wild-type (+/+), *nsy-1(ums8)/+* heterozygotes, and *nsy-1(ums8)/nsy-1(ums8)* homozygotes. *F08G5.6*::*GFP* was used as the wild-type strain. Data are the average of three replicates, each normalized to a control gene with error bars representing SEM, and are presented as the value relative to the average expression of the indicated gene in wild-type animals. * equals *P* < 0.05. There was no statistical difference in the levels of induction between the heterozygous and homozygous samples for either gene. For additional genes tested, see Figure S1A. (D) Immunoblot analysis of lysates from L4 larval stage animals of the indicated genotype using antibodies that recognize the doubly phosphorylated TGY motif of PMK-1 (p-PMK-1) and actin. Thirty μg of *nsy-1(ums8)*, *pmk-1(km25)* and *nsy-1(ag3)* total protein were loaded on the gel alongside a dilution series of wild-type template [15 μg, 20 μg, and 30 μg of total protein (left to right)] to control for the ability of the p-PMK-1 antibody to detect different concentrations of substrate. The arrow on the right highlights the PMK-1 band, which is absent in the *pmk-1(km25)* and *nsy-1(ag3)* mutants. The arrowheads on the left point to nonspecific bands. Data are representative of two biological replicates.

To explore further the physiological consequences of innate immune hyperactivation in *C. elegans*, we designed a forward genetic screen for endogenous activators of p38 MAP kinase PMK-1 signaling. The innate immune transcriptional reporter *F08G5.6*::*GFP* is induced during *P. aeruginosa* infection, and robustly by the anti-infective xenobiotic R24, in a manner dependent on the p38 MAPK PMK-1 pathway (Pukkila-Worley *et al.* 2012, 2014). We therefore reasoned that a screen for dominant activators of *F08G5.6*::*GFP* would identify mutations that cause constitutive activation of the p38 MAPK pathway. We screened the F1 progeny of mutagenized *F08G5.6*::*GFP* animals, and identified a single mutant allele, *ums8*, from approximately 170,000 mutagenized haploid genomes. Following both 1X and 2X outcrosses to wild-type N2 animals, we pooled F2 recombinants that were homozygous for the mutant phenotype, and sequenced the genomes of these samples using next-generation sequencing technology (Sarin *et al.* 2008; Minevich *et al.* 2012). This study revealed that the *ums8* mutant contained a G→A missense mutation in the coding region of *nsy-1* that resulted in the substitution of a strongly conserved Arg^246^ to Gln^246^ (Figure 2A). NSY-1/ASK1 is a conserved MAPKKK that acts upstream of the p38 MAPK PMK-1 to regulate immune and stress responses in *C. elegans* (Kim *et al.* 2002). We outcrossed the *ums8* strain a total of six times to wild-type N2, and confirmed that the R246Q mutation was present and *F08G5.6*::*GFP* expression was activated in the outcrossed strain.

To determine if the constitutive expression of the immune reporter *F08G5.6*::*GFP* in the *ums8* mutant is caused by the G→A mutation in the coding region of *nsy-1*, we used RNAi to knockdown the expression of *nsy-1* in the *ums8* mutant and found that this treatment suppressed *F08G5.6*::*GFP* induction (Figure 2B). Moreover, RNAi-mediated knockdown of *nsy-1* in wild-type animals did not cause *F08G5.6* induction (see qRT PCR data discussed in the next section). In addition, *F08G5.6*::*GFP* induction in the *nsy-1(ums8)* strain was suppressed by RNAi-mediated knockdown of *pmk-1*, the p38 MAPK that is downstream of *nsy-1* (Figure S2A). We also used qRT-PCR to confirm that *nsy-1(ums8)* is a dominant, gain-of-function allele that drives both the constitutive activation of the *F08G5.6*::*GFP* and the *F08G5.6* gene itself (Figure 2C). *F08G5.6* and *GFP* were induced to a similar degree in *nsy-1(ums8)/+* heterozygotes and in *nsy-1(ums8)/nsy-1(ums8)* homozygotes compared to wild-type controls that carried the transgene *F08G5.6*::*GFP* (+/+).

Consistent with our studies suggesting that *nsy-1(ums8)* is a gain-of-function allele of *nsy-1*, we performed an immunoblot analysis of protein lysates from *nsy-1(ums8*) animals and found that the *nsy-1(ums8)* animals had greater amounts of activated p38 MAPK PMK-1 than control animals (Figure 2D).

In summary, these data characterize a gain-of-function allele of the MAPKKK *nsy-1* that causes hyperactivation of the p38 MAPK pathway.

### The nsy-1(ums8) gain-of-function allele causes the constitutive activation of p38 MAPK PMK-1-dependent innate immune effectors

**Figure 3:**
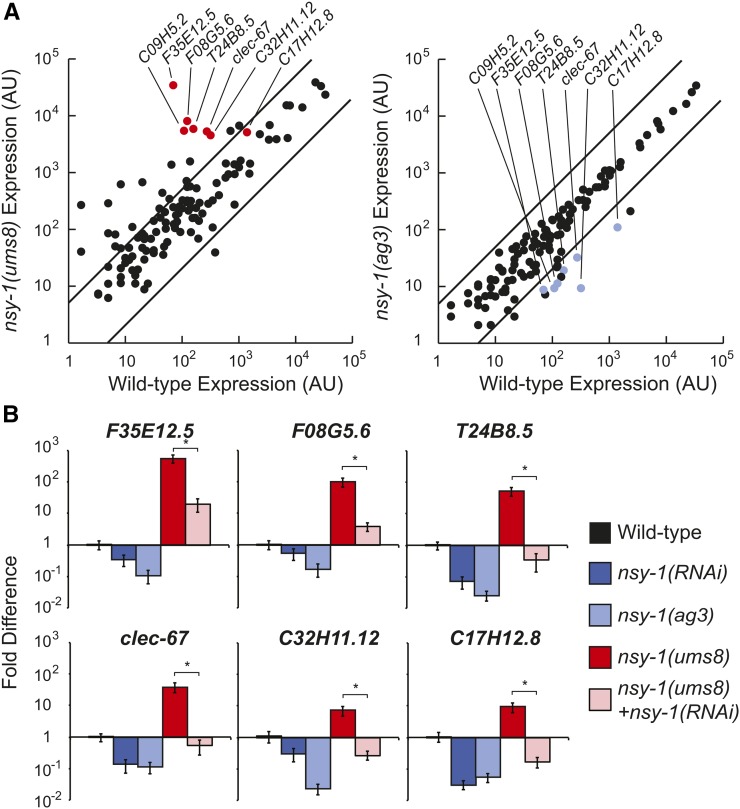
p38 MAPK-dependent putative immune effectors are constitutively activated in the *nsy-1(ums8)* mutant. (A) A scatter plot compares the expression of 118 *C. elegans* genes, which were analyzed using nanoString nCounter gene expression system in wild-type, *nsy-1(ums8)* and *nsy-1(ag3)* animals. Data are the average of two replicates for *nsy-1(ums8)*, and *nsy-1(ag3)*, and are from one sample for wild-type. The expression of each gene was normalized to the geometric mean of the expression of three control genes. Genes that are outside the two parallel lines on each graph are differentially regulated more than fivefold from the expression in wild-type animals. Genes that are previously characterized targets of the p38 MAPK PMK-1, and were strongly differentially regulated in this experiment, are highlighted. See Table S1 for the expression levels of all the genes. (B) qRT-PCR was used to study the expression of six putative immune effectors in RNAi-treated, mixed-stage animals of the indicated genotypes. All animals were grown on the RNAi bacteria feeder strain HT115 expressing the empty vector L4440, except for the two indicated samples that were exposed to bacteria expressing the *nsy-1(RNAi)* construct. The location on the scatter plots of these genes is indicated in (A) with red (left) and blue (right) dots. Data are the average of three replicates, each normalized to a control gene with error bars representing SEM, and are presented as the value relative to the average expression of the indicated gene in wild-type animals. * equals *P* < 0.05.

To determine if the basal expression of other genes was hyperactivated in the *nsy-1(ums8)* allele, we used the nanoString nCounter system to compare the expression profile of 118 innate immune and stress response genes in *nsy-1(ums8)* gain-of-function, *nsy-1(ag3)* loss-of-function and wild-type animals when the strains were feeding on the normal laboratory food source *E. coli* OP50 (Figure 3A). Of the 118 genes in this codeset, 24 were transcriptionally upregulated at least fivefold in *nsy-1(ums8)* mutants compared to wild-type animals (Table S1). Seven of the 24 genes most strongly upregulated in the *nsy-1(ums8)* gain-of-function allele were among the 12 most strongly reduced in the *nsy-1(ag3)* loss-of-function allele (*F08G5.6*, *F35E12.5*, *C09H5.2*, *T24B8.5*, *clec-67*, *C32H11.12*, and *oac-6*, Figure 3A and Table S1). Indeed, this group of seven genes includes *F08G5.6* and five other putative immune effectors (*F35E12.5*, *C09H5.2*, *T24B8.5*, *C32H11.12* and *clec-67*) that are previously characterized targets of the p38 MAPK PMK-1 (Table S1) (Troemel *et al.* 2006). Of note, the codeset included four additional genes whose basal or pathogen-induced expression requires the p38 MAPK PMK-1 (*F49F1.6*, *C17H12.8*, *C32H11.1*, *K08D8.5*, *lys-2*), and each of these genes was induced at least twofold in the *nsy-1(ums8)* mutants, and repressed at least fourfold in the *nsy-1(ag3)* mutants (Table S1). In addition, we previously used this codeset to identify 13 genes whose induction by the immunostimulatory anti-infective xenobiotic R24 was dependent on the p38 MAPK PMK-1 (Pukkila-Worley *et al.* 2014). Nine of these 13 genes were upregulated more than fivefold, and all of these genes were induced at least twofold in the *nsy-1(ums8)* gain-of-function mutant (Table S1). Thus, the *nsy-1(ums8)* gain-of-function allele drives the constitutive activation of genes whose basal, pathogen-induced or R24-induced expression is dependent the p38 MAPK pathway.

We confirmed these data in three biological replicate samples using qRT-PCR to study six putative immune effectors that were differentially regulated in the nanoString analysis and are known targets of the p38 MAPK pathway. All six genes were transcriptionally repressed in *nsy-1(RNAi)* and *nsy-1(ag3)* loss-of-function mutants, and were upregulated in the *nsy-1(ums8)* allele (Figure 3B). RNAi-mediated knockdown of *nsy-1* partially suppressed the constitutive activation of these six putative immune effectors in the *nsy-1(ums8)* allele (Figure 3B). In addition, we found that *nsy-1(ums8)/+* heterozygotes caused the induction of three p38 MAPK-dependent immune effectors to levels equivalent to that in *nsy-1(ums8)* homozygous animals (Figure S1A), as we observed for *F08G5.6* (Figure 2C). Of note, we also exposed the *nsy-1(ums8)* mutant to R24, and found that *F08G5.6*::*GFP* expression was markedly increased compared to untreated *nsy-1(ums8*) animals, suggesting that the effects of R24 and the *nsy-1* gain-of-function allele on gene expression are additive (Figure S1B). Consistent with this observation, we previously found that R24 also further enhanced the expression of *P. aeruginosa* immune effectors that are induced during bacterial infection (Pukkila-Worley *et al.* 2012).

Previous whole genome, transcriptome profiling analyses have identified a group of putative immune effectors whose basal expression is strongly repressed in *pmk-1(km25)* mutants (Troemel *et al.* 2006). The term basal regulation has been used in this context to describe the expression of genes in animals that are growing on the laboratory food source (*E. coli* OP50) and is used to distinguish from the pathogen-induced expression of putative immune effectors that is observed during microbial infection. In addition to controlling the basal expression of putative immune effectors, a number of studies have demonstrated that the p38 MAPK PMK-1 is also required for the pathogen-induced expression of putative immune effectors (Troemel *et al.* 2006; Bolz *et al.* 2010; Pukkila-Worley *et al.* 2012). It also is clear, however, that there is a group of genes, the identities of which have not been comprehensively defined, that require the p38 MAPK PMK-1 pathway for their induction, but not for their basal expression. The nanoString data of *nsy-1(ums8)* mutant indicates that this gain-of-function allele can be used to identify this group of innate immune effectors. Of the 24 genes in our codeset that were most strongly induced in the *nsy-1(ums8)* mutant, 17 were not dependent on NSY-1 for their basal expression—a group which includes both pathogen and stress-response genes (Table S1).

It is also interesting to note that genes involved in the detoxification of small molecule toxins, such as cytochrome P450s (CYPs), glutathione-s-transferases (GSTs), and UDP-glucuronosyltransferases (UDPs), were not among those transcriptionally upregulated in the *nsy-1(ums8)* gain-of-function allele (Table S1). We previously observed that the anti-infective xenobiotic R24 caused the robust induction of these gene classes, in addition to genes involved in the response to pathogens (Pukkila-Worley *et al.* 2012). Unlike the induction of antimicrobial immune effectors, however, the upregulation of detoxification genes by R24 was not dependent on the p38 MAPK PMK-1 pathway (Pukkila-Worley *et al.* 2014). Together with the findings in this study, these data argue that p38 MAPK innate immune hyperstimulation itself does not lead to the induction small molecule detoxification responses, and that other cellular mechanisms are engaged to recognize and respond to xenobiotic toxins in *C. elegans*.

### Endogenous hyperactivation of the p38 MAPK PMK-1 pathway protects nematodes from bacterial infection, but is toxic to C. elegans

Stimulation of the p38 MAPK pathway by the small molecule xenobiotic R24 protects *C. elegans* from bacterial infection by stimulating immune effector expression through the p38 MAPK pathway (Pukkila-Worley *et al.* 2012, 2014). Immune hyperactivation in this context has negative physiological consequences to nematodes developing under normal laboratory conditions (Figure 1). We therefore hypothesized that endogenous hyperactivation of the p38 MAPK pathway, by means of a gain-of-function mutation in the MAPKKK NSY-1, would display similar phenotypes as worms treated with R24.

**Figure 4:**
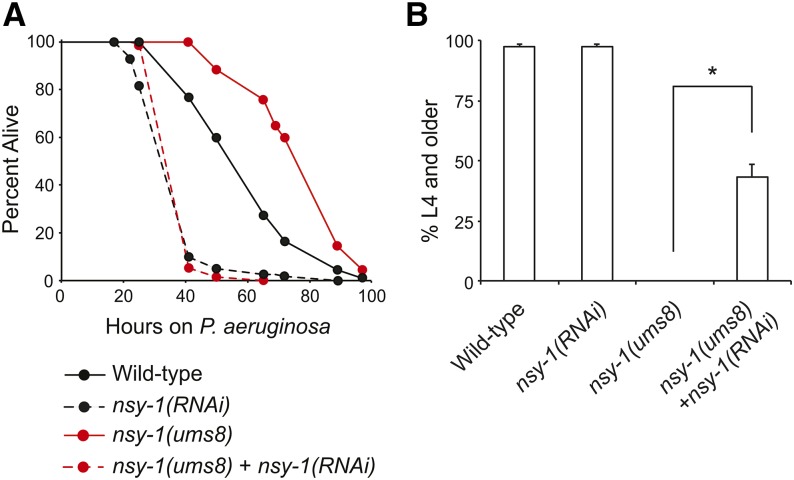
Endogenous hyperactivation of p38 MAPK innate immune responses protects nematodes from bacterial infection and delays the development of wild-type nematodes. In both (A) and (B), wild-type animals were grown on RNAi bacteria expressing the empty vector L4440 (Wild-type), or a construct designed to knockdown *nsy-1* [*nsy-1(RNAi)*]. *nsy-1(ums8)* animals, were grown in parallel on RNAi bacteria expressing the empty vector L4440 [*nsy-1(ums8)*], or a construct designed to knockdown *nsy-1* [*nsy-1(ums8)* + *nsy-1(RNAi)*]. (A) *P. aeruginosa* pathogenesis assays were performed on RNAi-treated animals of the indicated genotypes. The difference in *P. aeruginosa* susceptibility between wild-type and *nsy-1(ums8)* animals is significant, as is the survival difference between *nsy-1(ums8)* and *nsy-1(ums8)* + *nsy-1(RNAi)* (*P* < 0.001). Data are representative of two biological replicates. The sample sizes for this experiment are: wild-type (115), *nsy-1(RNAi)* (122), *nsy-1(ums8)* (111), and *nsy-1(ums8)* + *nsy-1(RNAi)* (133) (B) The development of RNAi-treated animals of the indicated genotypes to the L4 larval stage or older was recorded. The data are the average of three plates, with error bars showing the standard deviation between plates. The sample sizes for this experiment are: wild-type + L4440 (1,200), wild-type + *nsy-1(RNAi)* (1179), *nsy-1(ums8)* + L4440 (956), and *nsy-1(ums8)* + *nsy-1(RNAi)* (1010). Data are representative of two biological replicates. * *P* < 0.001.

We tested the susceptibility of *nsy-1(ums8)* gain-of-function mutant animals to *P. aeruginosa* infection compared to wild-type animals, and found that *nsy-1(ums8)* animals were resistant to killing by *P. aeruginosa* (Figure 4A and Figure S3). RNAi-mediated knockdown of *nsy-1* suppressed the resistance phenotype of *nsy-1(ums8)* animals (Figure 4A). Interestingly, the development of *nsy-1(ums8)* larvae was markedly delayed compared to wild-type controls, which was quantified by determining the number of mutant and wild-type animals that reached the L4 stage from synchronized eggs after 3 d of incubation at 20° (Figure 4B). Knockdown of *nsy-1* in *nsy-1(ums8)* animals partially suppressed this phenotype (Figure 2B and Figure 4B). We also observed that *nsy-1(ums8)* animals had markedly smaller brood sizes than wild-type controls (Figure S2B), data that are reminiscent of our observations with the immunostimulatory xenobiotic R24 (Pukkila-Worley *et al.* 2012). In addition, RNAi-mediated knockdown of *pmk-1* also suppressed the delayed development of *nsy-1(ums8)* animals (Figure S2C).

In this study, we provide direct evidence from two forward genetic screens that aberrant stimulation of p38 MAPK PMK-1-mediated innate immune responses via a gain-of-function mutation in *nsy-1* or the exogenous addition of the anti-infective R24 drives immune responses that are protective during infection, but are toxic to nematodes under normal growth conditions. It is interesting to note that the alleles with the most penetrant Xts phenotypes all had hypomorphic mutations in the known components of the p38 MAPK pathway. Of note, a forward genetic screen for p38 MAPK pathway components also did not identify mutations in any gene that functions upstream of *tir-1* (Shivers *et al.* 2010). Together, these data suggest that the p38 MAPK signaling cassette receives multiple inputs to regulate protective host responses in *C. elegans*.

In addition to promoting clearance of invading pathogens, an emerging body of literature has established that host systems also function to promote tolerance to infection, thereby reducing the negative impact that infection can have on organismal fitness (Ayres and Schneider 2008; 2009). Conceptually, host tolerance mechanisms function to mitigate the damage caused by the pathogen and also limit collateral injury associated with immune activation (Ayres and Schneider 2012; Medzhitov *et al.* 2012). Our studies of the immunostimulatory xenobiotic R24 and the *nsy-1(ums8)* gain-of-function allele suggest that host tolerance mechanisms may function in nematodes to limit the immunopathology associated with aberrant p38 MAPK activation and the ensuing hyperproduction of immune effectors. In *C. elegans*, the unfolded protein response (UPR) is one such mechanism that has been shown to promote tolerance during immune activation (Richardson *et al.* 2010; Sun *et al.* 2011). The IRE1-XBP1/Hac1 branch of the UPR is required to handle the physiological stress associated with an increase in p38 MAPK PMK-1 activity, which was conferred experimentally by exposure to *P. aeruginosa* or through RNAi-mediated knockdown of the MAPK phosphatase *vhp-1*, a negative regulator of PMK-1 (Richardson *et al.* 2010; Mizuno *et al.* 2004; Kim *et al.* 2004). Consistent with these data, we found that *xbp-1(zc12)* mutants are dramatically susceptible to the toxic effects of the immunostimulatory xenobiotic R24 in a developmental assay compared to control animals (Figure S4). Thus, there are likely at least two mechanisms in *C. elegans* to ensure immune homeostasis and promote tolerance during infection. The UPR functions to protect the host from the ER stress, which occurs as a consequence of p38 MAPK activation (Richardson *et al.* 2010). In addition, the isolation of a gain-of-function allele in *nsy-1* argues that p38 MAPK PMK-1-mediated transcriptional responses are controlled via upstream mechanisms that negatively regulate immune activation at the level of NSY-1.

The human homolog of NSY-1, ASK1, regulates p38 activity through at least three conserved protein domains: a central serine-threonine kinase domain and two coiled-coil domains in the N and C termini, respectively (Figure 2A) (Bunkoczi *et al.* 2007). The N-terminal domain binds thioredoxin (TRX), which inhibits the function of NSY-1. Interestingly, the gain-of-function *ums8* mutation, in which the negatively charged Arg is substituted for Gln, an uncharged amino acid with a large polar side chain, is located in a strongly conserved region, eight amino acids upstream of the region in NSY-1 predicted by homology to encode this N-terminal regulatory domain (Figure 2A). We hypothesize that the activity of *C. elegans* NSY-1 is normally controlled by a negative regulatory factor, which binds to the N-terminal coiled-coil domain. Thus, disruption of this region by the *ums8* mutation could account for the constitutive activation of p38 MAPK immune effectors resulting in a pathogen resistance phenotype and toxicity to developing nematodes. There are five thioredoxin (TRX) homologs in *C. elegans* and it will be interesting to determine if any of these genes function as negative regulators of NSY-1.

## Supplementary Material

Supporting Information
