# Supplementary material for: Aberrant Activation of p38 MAP Kinase-Dependent Innate Immune Responses Is Toxic to Caenorhabditis elegans
Source: G3 (Bethesda). 2016 Jan 27;6(3):541–9. doi: 10.1534/g3.115.025650 (PMC4777117; doi:10.1534/g3.115.025650)
Supplement: Supporting Information [file supp_6_3_541__index.html]

Aberrant Activation of p38 MAP Kinase-Dependent Innate Immune Responses Is Toxic to Caenorhabditis elegans — Supporting Information 

# Aberrant Activation of p38 MAP Kinase-Dependent Innate Immune Responses Is Toxic to *Caenorhabditis elegans*

## Supporting Information for Cheesman *et al.*, 2016

**Files in this Data Supplement:**

- Figure S1 - The *nasy-1(ums8)* gain-of-function allele causes the induction of p38 MAPK dependent putative immune effectors in a manner that is synergistic with R24. (.eps, 6,560 KB)
- Figure S2 - RNAI-mediated knockdown of *pmk-1* suppresses *F08G5.6::GFP* activation and the delayed development of the *nsy-1(ums8)* mutant. (.eps, 19,816 KB)
- Figure S3 - *nsy-1(ums8)* gain-of-function animals are resistant to killing by *P. aeruginosa*. (.eps, 744 KB)
- Figure S4 - XBP-1 is required for development in the presence of the immunostimulatory xenobiotic R24. (.eps, 1,465 KB)
- Table S1 - Relative Expression of the 118 Genes in the nanoString nCounter Gene Expression Analysis. (.xlsx, 21 KB)
